# Wavelet-based U-shape network for bioabsorbable vascular stents segmentation in IVOCT images

**DOI:** 10.3389/fphys.2024.1454835

**Published:** 2024-08-15

**Authors:** Mingfeng Lin, Quan Lan, Chenxi Huang, Bin Yang, Yuexin Yu

**Affiliations:** ^1^ Henan Key Laboratory of Cardiac Remodeling and Transplantation, Zhengzhou Seventh People’s Hospital, Zhengzhou, China; ^2^ School of Informatics, Xiamen University, Xiamen, China; ^3^ Department of Neurology and Department of Neuroscience, The First Affiliated Hospital of Xiamen University, School of Medicine, Xiamen University, Xiamen, China; ^4^ Fujian Key Laboratory of Brain Tumors Diagnosis and Precision Treatment, Xiamen, China

**Keywords:** deep learning, medical image processing, bioabsorbable vascular stent, wavelet transform, intravascular optical coherence tomography

## Abstract

**Background and Objective:**

Coronary artery disease remains a leading cause of mortality among individuals with cardiovascular conditions. The therapeutic use of bioresorbable vascular scaffolds (BVSs) through stent implantation is common, yet the effectiveness of current BVS segmentation techniques from Intravascular Optical Coherence Tomography (IVOCT) images is inadequate.

**Methods:**

This paper introduces an enhanced segmentation approach using a novel Wavelet-based U-shape network to address these challenges. We developed a Wavelet-based U-shape network that incorporates an Attention Gate (AG) and an Atrous Multi-scale Field Module (AMFM), designed to enhance the segmentation accuracy by improving the differentiation between the stent struts and the surrounding tissue. A unique wavelet fusion module mitigates the semantic gaps between different feature map branches, facilitating more effective feature integration.

**Results:**

Extensive experiments demonstrate that our model surpasses existing techniques in key metrics such as Dice coefficient, accuracy, sensitivity, and Intersection over Union (IoU), achieving scores of 85.10%, 99.77%, 86.93%, and 73.81%, respectively. The integration of AG, AMFM, and the fusion module played a crucial role in achieving these outcomes, indicating a significant enhancement in capturing detailed contextual information.

**Conclusion:**

The introduction of the Wavelet-based U-shape network marks a substantial improvement in the segmentation of BVSs in IVOCT images, suggesting potential benefits for clinical practices in coronary artery disease treatment. This approach may also be applicable to other intricate medical imaging segmentation tasks, indicating a broad scope for future research.

## 1 Introduction

Coronary artery disease (CAD) is a leading cause of mortality in individuals with cardiovascular diseases (Mehvari et al., 2024). Currently, stent implantation represents an effective nonsurgical intervention for managing CAD that is capable of dilating narrowed vessels and mitigating the recurrence of vascular obstruction posttreatment (Ullah et al., 2023). Metal stents are the most commonly utilized; however, they may induce stent thrombosis (Changal et al., 2021). In contrast, bioabsorbable vascular stents (BVSs), which are absorbable and harmless, have emerged as the optimal alternative to metal stents. Intravascular optical coherence tomography (IVOCT) is an imaging modality that can depict the cross-sectional structure of arteries with high resolution. Given that BVSs are invisible in X-ray angiography and that their material composition results in low spatial resolution and blurriness in coronary angiography (Arat et al., 2018), IVOCT is prevalently selected for assessing the quality of stent deployment during surgery and for inspecting restenosis (recurrent vascular obstruction) during follow-up evaluations. Segmentation of stent struts in IVOCT images is crucial for assisting clinicians in objectively assessing stent deployment, tissue coverage, and the burden of restenosis. Manual segmentation of stent struts in IVOCT images is impractical, as a single IVOCT pullback may contain thousands of stent struts, making it time-consuming and inefficient for experts to identify stents from medical images. Hence, the development of an expert system for the automatic segmentation of stents is necessary to provide quantitative data within the timeframe of surgical procedures.

To date, numerous researchers have embarked on studies concerning the automatic segmentation of vascular stents employing image processing techniques among others. Wang et al. (2018) ventured into stent strut detection in intravascular ultrasound images by extracting Haar-like features, employing both cascaded AdaBoost and SVM classifiers for training data to achieve stent detection outcomes. However, this SVM-based method incurs significant computational overhead as the sample size increases. Moreover, its detection robustness is compromised when stents are incomplete, geometrically irregular, or embedded into the lumen, thereby offering limited assistance in helping physicians to assess the level of stent deployment. Cao et al. (2018) introduced a Bioabsorbable Vascular Stent (BVS) struts detection method employing a Region-based Fully Convolutional Network (R-FCN), wherein regions of interest in IVOCT images were extracted using an Region Proposal Network (RPN) module, and FCN was utilized to identify regions containing stent struts. Although this method enhances detection robustness across various scenarios, there remains room for improvement in its effectiveness. Bologna et al. (2019) delineated a three-step process for identifying stent struts in images, initially employing intensity thresholding using the 0.85 quantile of the pixel intensity distribution, followed by a flood fill operation to close holes in the binary image, and ultimately extracting BVS struts from IVOCT images through Boolean subtraction. Duda et al. (2022) commenced with a series of preprocessing steps on IVOCT images, incorporating contrast-limited adaptive histogram equalization and the Otsu threshold method (Sha et al., 2016), and concluded with Canny edge detection for strut detection.

With the rapid advancement of deep learning, methods exhibiting superior performance in the detection or segmentation of BVS in IVOCT images are emerging in the research landscape (Etehadtavakol et al., 2024). Zhou et al. (2019) proposed a U-Net-based BVS segmentation approach, wherein they modified the U-Net architecture to better suit biomedical IVOCT images, featuring five downsampling modules and four upsampling modules. Lau et al. (2021) integrated MobileNetV2 and DenseNet121 with U-Net to create a hybrid Encoder-Decoder Network, which enhanced the speed and accuracy of vascular scaffold segmentation beyond that of a singular U-Net structure. Huang et al. (2021) improved the U-Net structure by incorporating an attention layer to focus on significant areas within IVOCT images and utilized a Dilated convolution module (DCM) to attain a larger receptive field. This method also employed semi-supervised learning to address the issue of labor-intensive and time-consuming BVS annotation. Han et al. (2023) introduced a multiple attention convolutional model akin to the yolov5 architecture for stent struts detection, integrating squeeze and excitation (SE) attention (Hu et al., 2018) with the convolutional block attention module (CBAM) (Woo et al., 2018) into their detection network to achieve superior detection outcomes.

Overall, traditional machine learning methods and some conventional image processing techniques underperform in the task of segmenting or detecting bioresorbable vascular scaffolds in IVOCT images, suffering from low credibility of detection results and poor robustness in complex stent scenarios, such as stent deformation. Although some deep learning-based segmentation methods have managed to speed up segmentation and enhance performance, they fail to fully recognize features in IVOCT images, such as blood artifacts. These methods are not yet suitable for inclusion in expert systems that assist physicians with diagnoses, where high accuracy and reliability are needed.

Given the limitations outlined above, to achieve improved segmentation results, we propose a Wavelet-Based U-shape network for segmentation. Segmenting stent struts in high spatial resolution IVOCT images is often limited by artifacts that obscure the clear delineation of scaffolds from these distractions. Traditional convolutional networks struggle to accommodate the interference of artifacts within the stent struts regions to be segmented, potentially leading to low accuracy in segmentation outcomes. Hence, we introduce a wavelet branch to integrate with the original Encoder structure, aiming to merge multi-dimensional features to enhance the perception of areas of interest. This wavelet branch processes the High Frequency (HF) part of the original image after undergoing a 2D Discrete Wavelet Transform, which captures clear detail and edge information more effectively. Compared to the single-branch Encoder used by Zhou et al. (2019), our dual-branch Encoder structure enables the model to focus more on the minute stent struts, thereby enhancing the segmentation results.

We designed and introduced a Wavelet Fusion Block to merge features from the original convolutional branch with those from the wavelet branch. This amalgamation leverages both the semantic features of the original images and the detailed edge characteristics from the wavelet branch to achieve superior segmentation results. Additionally, an Atrous Multi-scale Field Module (AMFM) is incorporated at the base of our proposed network to attain a larger receptive field, capturing texture and detail information across multiple scales. Atrous convolution, also known as dilated convolution, expands the receptive field without additional computational cost, enabling the network to learn multi-scale contextual information. Furthermore, we integrated an Attention Gate (AG) with the network to suppress feature responses in irrelevant background regions and enhance the feature response in the BVS area or regions of interest. With these modules, our proposed network’s generalization ability and segmentation performance are enhanced, and its adaptability and robustness to artifacts and noise in IVOCT images are improved to some degree. Overall, the contributions of this work are summarized as follows:

(1) A novel U-Net-based network is proposed, incorporating a wavelet branch with the convolutional branch to enhance the segmentation effectiveness for BVS in IVOCT images and improve adaptability to noise and artifacts.
(2) The Atrous Multi-scale Field Module (AMFM) is utilized to enable the network to learn multi-scale contextual feature information, enhancing the capability to capture long-range dependencies.
(3) A wavelet fusion block is designed for merging original and HF features, optimizing the semantic alignment and integration of dual-branch information.
(4) The Attention Gate (AG) is integrated with the level-by-level convolutional network, strengthening the feature response of the region of interest for better segmentation outcomes.

The remainder of the paper is organized as follows: Section 2 provides a brief overview of the related work. Section 3 introduces our proposed network along with other modules. Section 4 describes the evaluation metrics and experimental results. In Section 5, we offer a brief discussion of the work presented in this paper. Finally, Section 6 concludes the paper and outlines future research directions.

## 2 Related work

In the domain of medical image segmentation, since its introduction in 2015, the U-Net model (Ronneberger et al., 2015) has become a milestone methodology, widely applied to the automatic segmentation of various medical images. U-Net, through its unique symmetrical design of downsampling and upsampling, effectively captures the contextual information of images while maintaining sensitivity to details. This design has enabled U-Net to exhibit exceptional performance in processing medical images with complex structures, especially when there is a limited amount of labeled data. Subsequent studies have introduced various variants and improvements of the U-Net model to meet different medical image segmentation needs, further demonstrating the architecture’s strong adaptability and effectiveness.

Building on the foundation of U-Net, the Attention U-Net (Oktay et al., 2018) incorporates a novel attention module, known as the Attention Gate (AG). This model integrates AG into the level-by-level U-Net structure, specifically, it modulates the feature maps from one upsampling operation and the parallel feature maps from skip connections through the AG attention mechanism. This suppresses the attention to background areas, enabling the model to focus more on the foreground parts to be extracted. In the same year, ResUnet (Zhang et al., 2018) employs residual units in place of the basic convolutional blocks of the original U-Net structure. The advantage of this modification is that residual units are easier for the network to train and can integrate high-level and low-level information without degradation, allowing the region of interest to be identified at multiple scales. ResUnet++ (Jha et al., 2019), an enhancement of ResUnet aimed at colonoscopy image segmentation, incorporates an Atrous spatial pyramid pooling (ASPP) module that acts as a bridge between the encoder’s output and the decoder’s input to expand the receptive field. It also uses an SE module (squeeze and excitation block) to enhance the foreground awareness during the downsampling process of the model, though this method might result in the loss of some low-level detail information, reducing segmentation precision. As the transformer structure has been widely used for visual tasks, Swin-Unet (Cao et al., 2022) represents a hybrid model for medical image segmentation. This approach feeds image patches into a transformer-based U-Net network structure and combines skip connections to learn global semantic information that pure CNN-based networks might struggle to fully grasp. Although capable of capturing long-distance dependencies, this patch input method imposes limitations on image inputs, particularly for high spatial resolution medical images. Compared to the models mentioned above, our method not only enhances the ability of feature extraction but also improves robustness in noisy backgrounds, as evidenced by our extensive experiments where we achieved superior performance with a Dice coefficient of 85.10%, accuracy of 99.77%, sensitivity of 86.93%, and IoU of 73.81%, surpassing the results reported by previous studies.

In recent years, the integration of wavelet transform with the U-Net model has garnered widespread attention in the field of medical image segmentation. Incorporating wavelet transform within the U-Net architecture allows for effective multi-scale analysis of images, thereby better capturing and utilizing texture and detail information within the images. Aerial LaneNet (Azimi et al., 2018) is a symmetric FCNN model enhanced by wavelet; it views wavelet transform as a tool for extracting full-spectral information in the frequency domain and integrates it into the CNN. However, this method is specifically designed for lane marking in aerial imagery. CWNN (Gao et al., 2019), another model that combines CNN with wavelet transform, focuses on sea ice change detection from synthetic aperture radar (SAR) images. It introduces the dual-tree complex wavelet transform to improve the pooling layer, achieving more robust and reliable detection results, but the model is still limited to a specific use case and lacks generalizability. Wavesnet (Li and Shen, 2022) employs discrete wavelet transform (DWT) to extract image details during downsampling and uses Inverse DWT to restore detail information during upsampling. This symmetrical approach fits well with U-Net’s Encoder-decoder structure. Xnet (Zhou et al., 2023), by improving upon previous wavelet-integrated methods, designs a dual-branch for HF and LF images and uses feature fusion technology to perform dual-decoder output, selecting the optimal segmentation results. Furthermore, unlike Wavesnet (Li and Shen, 2022) and Xnet (Zhou et al., 2023), our model does not rely on offline wavelet transform or a computationally expensive dual encoder-decoder structure, making it more efficient and applicable to a broader range of medical imaging scenarios.

The introduction of wavelet transform not only enhances the model’s sensitivity to features at different frequencies but also improves its robustness in noisy backgrounds. This approach is particularly suitable for scenarios where texture information significantly impacts segmentation accuracy, such as boundary recognition or segmentation of fine structures. By merging U-Net’s deep feature extraction capabilities with the multi-scale analysis advantages of the wavelet transform, researchers have been able to develop more accurate and robust medical image segmentation models, providing more reliable support tools for clinical diagnosis and treatment.

## 3 Materials and methods

### 3.1 Overview

**FIGURE 1:**
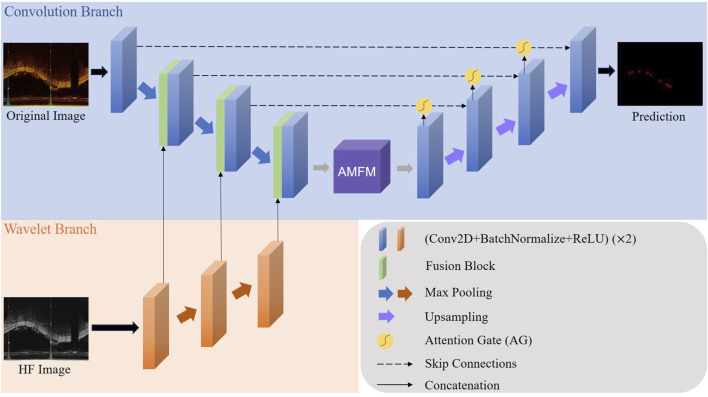
The whole architecture of our proposed model. The Convolution Branch and the Wavelet Branch represent the traditional encoder structure and our proposed wavelet-based encoder structure, respectively.

Our proposed model structure, as shown in Figure 1, is designed for a Wavelet-based U-shape convolutional network aimed at BVS segmentation in IVOCT images. The model principally comprises two branches: a convolutional branch, which serves as the plain U-Net encoder, and a wavelet branch that inputs HF features. Bridging these two branches, the key component is the Wavelet Fusion Module, which integrates the original features with HF features to form multi-dimensional features. Moreover, the model incorporates two major components: the Atrous Multi-scale Field Module (AMFM) and the Attention Gate (AG).

The AMFM enriches the model’s ability to capture a broader range of contextual information and long-distance dependencies by fusing multiscale information to achieve a larger receptive field. By feeding the feature maps from the encoder side and the parallel feature maps from the decoder side into the AG via skip connections, the response of unrelated areas is suppressed, while the response of regions of interest is enhanced, thus achieving better segmentation results. This strategic integration of components ensures that our model not only effectively handles the inherent challenges in IVOCT image segmentation, such as noise and artifacts but also improves the precision and robustness of segmentation outcomes.

### 3.2 Wavelet Transform

Intravascular optical coherence tomography (IVOCT) images, which are 2D high-spatial-resolution images, are discrete nonstationary signals containing rich information in both the frequency and spatial domains. The wavelet transform is an effective tool for capturing information within images while maintaining robustness to noise (Zavala-Mondragón et al., 2021). The 2D discrete wavelet transform (DWT) enables the decomposition of an image into one low-frequency component and three high-frequency components. The low-frequency component, referred to as LL, retains most of the semantic information of the image but with reduced resolution and less detail. The three high-frequency components, denoted as HL, LH, and HH, capture vertical, horizontal, and diagonal detail information, respectively.

For a 2D image X , we employ the 2D Discrete Wavelet Transform to decompose it into four parts, namely, LL, HL, LH, and HH:

$$X_{ij}=\left(↓2\right)\left(f_{ij}*X\right),\;\;i,j\in \left\{L,H\right\}$$ (1)

In this context, $f_{LL}$ represents the low-pass filter, while $f_{HL}$ , $f_{LH}$ and $f_{HH}$ represent to high-pass filters in the vertical, horizontal, and diagonal directions, respectively. The asterisk * denotes the convolution operation. Through the application of the 2D Discrete Wavelet Transform, the outputs obtained are $X_{LL}$ , $X_{HL}$ , $X_{LH}$ and $X_{HH}$ , which represent the LL, HL, LH, and HH components, respectively.

Taking the 1D Haar wavelet (Stanković and Falkowski, 2003) as an example, its low-pass filter $f_{L}$ and high-pass filter $f_{H}$ are defined as:

$$f_{L}=\frac{1}{\sqrt{2}}\left[\begin{array}{c} 1\\ 1 \end{array}\right],\;f_{H}=\frac{1}{\sqrt{2}}\left[\begin{array}{c} 1\\ -1 \end{array}\right]$$ (2)

Meanwhile, the 2D Haar wavelet low-pass filter $f_{LL}$ and the three high-pass filter $f_{HL}$ , $f_{LH}$ and $f_{HH}$ are defined as:

$$f_{LL}=f_{L}⊗f_{L}=\frac{1}{2}\left[\begin{array}{cc} 1 & 1\\ 1 & 1 \end{array}\right],\;f_{HL}=f_{H}⊗f_{L}=\frac{1}{2}\left[\begin{array}{cc} 1 & -1\\ 1 & -1 \end{array}\right]$$ (3)

$$f_{LH}=f_{L}⊗f_{H}=\frac{1}{2}\left[\begin{array}{cc} 1 & 1\\ -1 & -1 \end{array}\right],\;f_{HH}=f_{H}⊗f_{H}=\frac{1}{2}\left[\begin{array}{cc} 1 & -1\\ -1 & 1 \end{array}\right]$$ (4)

We define the LF component as the LL component, while the HF component is a combination of the HL, LH, and HH components, representing the details in various directions of the original image. This distinction is particularly crucial for segmenting stent struts, which are small in area and have detailed edges in the foreground. Our definitions of LF and HF are as follows:

LF=LL,HF=HL+LH+HH (5)

**FIGURE 2:**
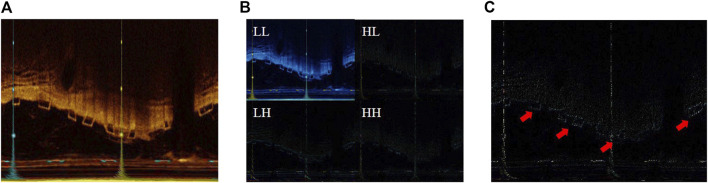
**(A)** The original IVOCT image; **(B)** the result of 2D DWT or visualization of LL, HL, LH, and HH components; **(C)** visualization of the HF components.

The LF and HF components are illustrated in Figure 2. Compared to the original image, the LF, which is the LL, is blurred and loses detailed information. On the other hand, the HF emphasizes detail information, significantly aiding in the precise localization of the BVS by the model.

### 3.3 Attention gate

In our proposed method, we have incorporated an Attention Gate (AG) mechanism, inspired by the Attention U-Net architecture (Oktay et al., 2018), aimed specifically at refining feature extraction for medical image segmentation tasks, particularly for the detection of stent struts. The AG module enables the model to focus on salient features relevant to the specific task at hand while suppressing the influence of unrelated background information. By selectively emphasizing important spatial regions and features within the input image, AG enhances the model’s ability to distinguish the area of interest from surrounding tissues and artifacts.

The core principle of the Attention Gate is to generate a gating signal that modulates the feature activation before the convolutional operations in the network. Through this, the two feature maps x and g will be modulated by two 1×1 convolutions respectively, followed by concatenation and activation with a ReLU function. Subsequently, the feature map is compressed into a single channel, and finally, an attention coefficient α , ranging from 0 to 1, is generated through a sigmoid activation function. This attention coefficient is then applied in an element-wise multiplication with the original feature map to produce a new feature map that highlights important areas. This process can be viewed as a gating mechanism that learns to weigh the importance of different features at different spatial locations, thereby allowing the network to focus more on relevant structures within the image. The AG operation does not require explicit external Region of Interest (ROI) cropping or sampling, making it an efficient and effective method to enhance the accuracy of segmentation results (due to the uniqueness of the gating feature map, which dynamically adjusts feature focus across the entire image without predefined zones).

**FIGURE 3:**
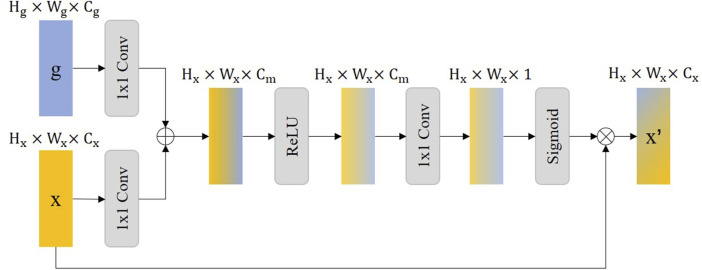
The illustration of the Attention Gate (AG), where the $C_{m}$ is equal to $C_{g}$ plus $C_{x}$ . ReLU represents the ReLU activation function.

In Figure 3, we illustrate that x represents the feature map in the decoder that needs to be upsampled, while g is the feature map in the encoder with the same resolution, used as a gating feature map to guide x towards paying more attention to important features. This gating feature map, being closer to the original input level, possesses more contextual information for reducing irrelevant feature responses. The feature map with attention $x^{\prime }$ is defined as follows:

$$\alpha =\sigma \left({f_{out}}^{1\times 1}\left(ReLU\left(f_{g}^{1\times 1}\left(g\right)⊕f_{x}^{1\times 1}\left(x\right)\right)\right)\right)$$ (6)

$$x^{\prime }=\alpha ⊗x$$ (7)

In the description, ⊕ denotes concatenation, ⊗ represents element-wise multiplication, ReLU stands for the Rectified Linear Unit, and $\sigma \left(\cdot \right)$ denotes the Sigmoid activation function, which is used to generate the attention coefficient α ranging from 0 to 1. The term $f_{\text{out}}^{1\times 1}$ refers to a 1×1 convolution employed to reduce the number of channels to 1, while $f_{\text{g}}^{1\times 1}$ and $f_{\text{x}}^{1\times 1}$ respectively indicate 1×1 transition convolutions used to unify the number of channels of the gating feature map and the feature map.

Integrating the AG into our model improves the accuracy of stent strut detection by enhancing feature contrast and detail resolution. Moreover, this approach can reduce false-positive predictions due to the difficulty of modeling the relationship between the stent struts and the surrounding tissue on a global scale. This results in more accurate and clinically useful segmentation results that are critical for assisting medical professionals in diagnosis and treatment planning.

### 3.4 Atrous multi-scale field module

Atrous convolution, also known as dilated convolution, aims to expand the filter’s receptive field without losing resolution, allowing the model to capture more contextual information without increasing the number of parameters or the amount of computation (Chen et al., 2017). Unlike standard convolution, which acts on adjacent pixels, atrous convolution introduces gaps in the input feature map, allowing the filter to cover a wider area of the input image. The expansion rate determines the spacing of each unit in the convolution kernel; An expansion rate of one indicates a regular convolution, while a higher expansion rate implies a wider range of inputs considered in each convolution step.

Accurate semantic information is crucial to the feature decoupling process. Therefore, we proposed the Atrous Multi-scale Field Module (AMFM) to expand the receptive field of the feature map in the lowest layer of the network through Atrous convolution to obtain more accurate contextual semantic information. Considering that a single Atrous convolution may lack generality, we use multiple dilated rates of Atrous convolution to capture multi-scale details to capture global semantic information.

**FIGURE 4:**
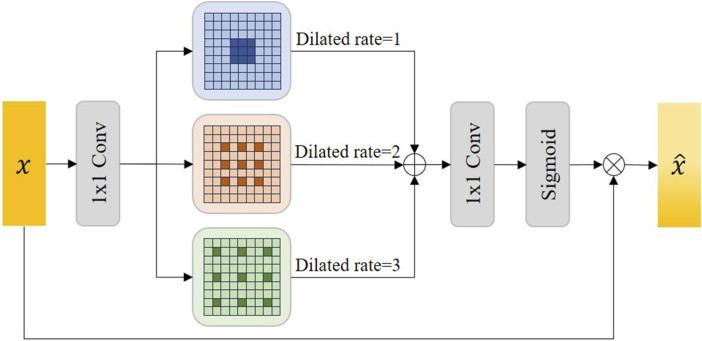
The illustration of our proposed Atrous Multi-scale Field Module (AMFM). Sigmoid represents the sigmoid activation function and 1×1 Conv denotes the transition convolution.

Figure 4 shows the details of the AMFM module we designed. In the AMFM module, the feature map x output by the encoder will first extracts important information through a 1×1 convolution, and then the feature map undergoes a 3×3 convolution with 3 different dilated rates, dilated rates are 1, 2, and 3 respectively. The purpose of this step is to expand the receptive field. The model can learn more semantic context information in IVOCT images. After atrous convolution, the three feature maps will be concatenated through a 1×1 transition convolution to change the number of channels. Finally, after activating the function by Sigmoid, the element-wise multiplication is executed with the original feature map x to obtain the final feature map $\hat{x}$ . In this process, the number of channels in the input feature map x will be the same as the number of channels in the output feature map $\hat{x}$ , but the output feature map $\hat{x}$ contains more semantic information. Moreover, it will capture more sufficient information for segmentation. We define this process as follows:

$$\hat{x}=x⊗\sigma \left(\overset{3}{\underset{i=1}{\sum }}f_{\text{d}=\text{i}}^{\;3\times 3}\left(f^{1\times 1}\left(x\right)\right)\right)$$ (8)

Where ⊗ stands for element-wise multiplication, $f_{d=i}^{\;3\times 3}\left(\cdot \right)$ stands for 3×3 atrous convolution, where d=i stands for dilated rate i , $f^{1\times 1}\left(\cdot \right)$ stands for 1×1 transition convolution. Note that the Σ represents the concatenation of the three feature maps together.

### 3.5 Wavelet fusion module

**FIGURE 5:**
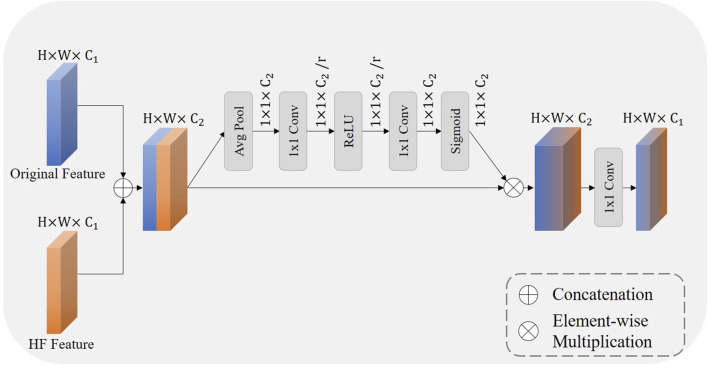
The illustration of our proposed wavelet fusion module, where r equals to 16 here.

To better combine the distinct characteristics of the original feature map and the HF feature map, we designed a wavelet fusion module, the structure of which is illustrated in Figure 5. The original feature map contains most of the semantic information of the original image but loses details and resolution during the downsampling process (Wang et al., 2023). On the other hand, the HF feature map, derived from the high-frequency components of the 2D DWT, encompasses the detail information of IVOCT images (Li et al., 2023). Through the wavelet fusion module, we can fuse features with different focal points to achieve a richer feature representation, thereby obtaining better segmentation results.

Within the wavelet fusion module, the two feature maps are first concatenated, which increases the number of channels. Inspired by the squeeze and excitation block (Hu et al., 2018), we also designed a similar channel attention mechanism. This results in a feature map that has been refined through an attention mechanism, enhancing performance in subsequent processing stages. Initially, global spatial information is compressed into a channel descriptor through global average pooling, facilitating a global understanding of the feature map. Subsequently, the inter-channel dependencies are captured through two layers of 1×1 convolution. This mechanism allows the network to focus on more informative features by adjusting the channel based on the importance learned for each channel, effectively recalibrating features based on their learned importance (Ghaffarian et al., 2021). The reduction ratio r is a parameter which represents the degree to which the number of channels is reduced during the compression step. By setting r , we control the balance between model complexity and the ability to capture channel-wise dependencies.

The recalibrated channel attention is then applied to the concatenated feature map through a multiplication operation. This operation selectively emphasizes features more relevant to the current task while suppressing less useful information, thereby enhancing the network’s representational capability. Finally, the channel count is reduced through a 1 × 1 transition convolution, which also adds non-linearity to the network. We define this process as follows:

$$F_{\mathrm{out}}=F_{m}⊗\left(\sigma \left(f^{1\times 1}\left(\mathrm{ReLU}\left(f^{1\times 1}\left(AvgPool\left(F_{m}\right)\right)\right)\right)\right)\right)$$ (9)

$$F_{m}=F_{1}⊕F_{2}$$ (10)

Where $F_{1}$ and $F_{2}$ are the input original feature map and HF feature map, respectively, $F_{m}$ is the concatenated feature map, and $F_{\mathrm{out}}$ is the final feature map after feature fusion. $\sigma \left(\cdot \right)$ represents the Sigmoid activation function.

### 3.6 Loss function

In the task of BVS segmentation in IVOCT images, since the shape of the segmentation target of the support pillar is usually a series of discontinuous small squares, the foreground takes a small proportion of the whole image. If the cross-entropy (CE) loss function is used, class unbalance leads to poor model optimization, resulting in poor segmentation. For this task, we choose to use the Dice coefficient loss function for model optimization (Milletari et al., 2016), which is defined as follows:

$$L_{\mathrm{dice}}=1-\frac{2\sum_{i=1}^{N}p_{i}q_{i}}{\sum_{i=1}^{N}p_{i}^{2}+\sum_{i=1}^{N}q_{i}^{2}}$$ (11)

Where N represents the total number of pixels, $p_{i}$ and $q_{i}$ represent the predicted label and the true label of pixel i .

## 4 Results

### 4.1 Experiment settings

#### 4.1.1 Dataset

The experimental data for this study were sourced from the Dongfang Hospital Affiliated to Tongji University. The dataset comprises 641 IVOCT images in the polar coordinate system, with a resolution of 527×704 . Each image was annotated by experienced clinicians. The ground truth data were generated through a rigorous process that involved the careful examination of each image to identify and delineate the regions of interest with high precision. We divided these data into training and testing sets at an 8:2 ratio. To prevent overfitting and enhance segmentation performance (Buslaev et al., 2020), we employed data augmentation techniques. Specifically, we applied random adjustments to hue, saturation, and value to simulate different camera settings, thereby enriching the diversity of our images. Additionally, we utilized random shift, scale, and rotation augmentations to further increase the variability within the dataset. Moreover, we included random horizontal and vertical flips.

#### 4.1.2 Model implementation and metrics

This model uses the PyTorch 1.7.0 framework and the NVIDIA Tesla V100 SXM2 with 32 GB memory as the training tool. We set the batch size to 4, the epoch to 100, the initial learning rate to 0.001, and use AdamW to optimize the training process. At the same time, we use the step learning rate adjustment strategy, that is, the learning rate decreases by 50% every 20 rounds.

We used Dice coefficient (Dice), Accuracy, Sensitivity, and Intersection Over Union (IoU) as evaluation indicators, and their definitions are as follows:

$$Dice=\frac{2|A\cap B|}{\left|A|+|B\right|}=\frac{2TP}{2TP+FP+FN}$$ (12)

$$Accuracy=\frac{TP+TN}{TP+TN+FP+FN}$$ (13)

$$Sensitivity=\frac{TP}{TP+FN}$$ (14)

$$IoU=\frac{\left|A\cap B\right|}{\left|A\cup B\right|}=\frac{TP}{TP+FP+FN}$$ (15)

where TP, TN, FP, and FN denote the number of true positives, true negatives, false positives, and false negatives. A denotes the segmentation result and B denotes the Ground truth.

### 4.2 Comparison with state-of-the-art methods

To demonstrate the superiority of our proposed network in BVS segmentation within IVOCT images, we conducted comparative experiments with other state-of-the-art models, including U-Net (Ronneberger et al., 2015), FCN (Liang-Chieh et al., 2015), Attention U-Net (Oktay et al., 2018), ResUnet (Zhang et al., 2018), R2U-Net (Alom et al., 2018), ResUnet++ (Jha et al., 2019), HRNet-18 (Sun et al., 2019), Swin-Unet (Cao et al., 2022), DuckNet (Dumitru et al., 2023), DconnNet (Yang and Farsiu, 2023), and XNet (Zhou et al., 2023). The experimental results are presented in Table 1.

**TABLE 1:** Comparison with state-of-the-art methods, the best experimental results are shown in bold.

Methods	Years	Dice (%)	Accuracy (%)	Sensitivity (%)	IoU (%)
U-Net	2015	80.53	99.24	80.86	67.25
FCN	2014	79.10	99.68	81.28	65.43
Attention Unet	2018	84.06	99.75	84.97	71.81
ResUnet	2018	83.45	99.75	83.19	71.61
R2U-Net	2018	82.19	99.73	81.59	69.77
ResUnet++	2019	83.35	99.75	83.08	71.45
HRNet-18	2019	84.02	99.76	82.67	72.46
Swin-Unet	2022	60.77	99.40	64.62	43.64
DuckNet	2023	81.79	99.74	78.22	69.19
DconnNet	2023	83.53	99.73	86.45	71.71
XNet	2023	82.56	99.76	82.57	70.84
Ours	2024	**85.10**	**99.77**	**86.93**	**73.81**

From Table 1, we observe that traditional networks such as U-Net and FCN exhibit average performance in BVS segmentation in IVOCT images, with Dice coefficients of approximately 80% and IoUs between 65%–70%. Models incorporating attention modules or residual units, such as Attention U-Net, ResUnet, and ResUnet++, show some improvements in Dice coefficients and other metrics. Surprisingly, the recently proposed Swin-Unet performed the worst in this task, achieving only a 60.77% Dice coefficient. Xnet, which uses a dual-branch for HF images and LF images as inputs, displayed segmentation effectiveness similar to ResUnet. Our proposed network outperformed these models, achieving the highest Dice coefficient of 85.10%, an improvement of 1.24% over the second-best score of 84.06%, and showed enhancements in IoU, accuracy, and sensitivity to 73.81%, 99.77%, and 86.93%, respectively. Notably, our model scored the highest in all evaluation metrics, indicating significant improvements and the best segmentation performance.

**FIGURE 6:**
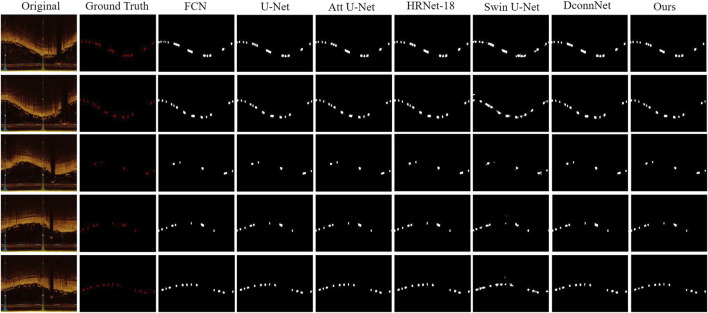
Visual comparison between our proposed method and state-of-the-art networks. It offers a side-by-side analysis of the segmentation results produced by our innovative approach and those of the current leading methods in the field.

In Figure 6, we visualized the segmentation results for a qualitative comparison. Swin-Unet, which had the lowest performance metrics, resulted in the poorest segmentation of BVS, potentially leading to false-positive segments and poor edge delineation of stent struts, a lack of connectivity and distortion of the original shape of the stent struts. This could minimally assist or even interfere with clinical decision-making and procedures. In contrast, our model achieved the best segmentation results, capturing the edge details of the stent struts well while maintaining the distribution of stent struts across the IVOCT images. For instance, DconnNet, which focuses on segment connectivity, tends to merge closely situated stent struts into a larger entity, whereas our model can correctly distinguish between individual stent struts.

### 4.3 Ablation studies

#### 4.3.1 Ablation on model components

To illustrate the role of the modules within our model, we conducted ablation studies on the Attention Gate (AG) and Atrous Multi-scale Field Module (AMFM), as shown in Table 2.

**TABLE 2:** Ablation study results of the AG and AMFM.

Method	AG	AMFM	Dice	Accuracy	Sensitivity	IoU
Baseline	×	×	83.10	99.74	83.76	71.08
	×	✓	84.73	99.76	85.81	73.46
	✓	×	84.59	99.76	86.16	73.29
	✓	✓	**85.10**	**99.77**	**86.93**	**73.81**

The best experimental results are shown in bold.

Commonly, the inclusion of these two modules improved the evaluation metrics of the baseline model, but their enhancements focused on different aspects. When the AG was added to the baseline model, there was a greater increase in sensitivity compared to AMFM. This is because AG focuses on reducing the response to unimportant features to decrease false-positive segmentation. Conversely, when AMFM was added to the baseline model, there was a greater improvement in IoU compared to AG. This is attributed to the AMFM module’s emphasis on expanding the model’s receptive field and enhancing its ability to capture long-distance dependencies, thus improving segmentation by modeling the differences between the background and foreground at a global scale. The model incorporating all modules performed optimally because the inclusion of AG and AMFM enhanced the model’s feature extraction capabilities from multiple perspectives, yielding the best results.

#### 4.3.2 Ablation on wavelets bases

To determine the optimal wavelet for BVS segmentation in IVOCT images, we experimented with different wavelet bases in the 2D Discrete Wavelet Transform (DWT). We tested several classic wavelets, including Haar, Daubechies 2, Daubechies 3, Coiflets 2, Symlets 2, Meyer, Discrete Meyer (Dmey), Biorthogonal 1.5, and Biorthogonal 2.4 wavelets. According to the results presented in Table 3, we found that the Biorthogonal 2.4 wavelet demonstrated the best performance. Consequently, all other experiments in our model were based on the 2D DWT using the Biorthogonal 2.4 wavelet. Some wavelet bases, such as Biorthogonal wavelets, are more suitable for edge detection because they can more effectively capture the high-frequency components of the image, which is particularly important for the representation of edges and details. This also explains why the choice of Biorthogonal 2.4 wavelet led to the best results. On the other hand, wavelet bases like Coiflets offer a smoother effect, which may result in weaker detail extraction capabilities, further explaining why the performance of Coiflets wavelet was not optimal.

**TABLE 3:** Ablation study results on wavelet bases.

Wavelets	Dice (%)	Accuracy (%)	Sensitivity (%)	IoU (%)
Haar	84.57	99.76	85.67	73.26
Meyer	84.46	99.76	85.29	73.09
Symlets 2	84.43	99.76	85.34	73.05
Coiflets 2	83.84	99.75	84.43	71.67
Discrete Meyer	84.35	99.75	85.98	72.94
Daubechies 2	84.17	99.76	85.50	72.66
Daubechies 3	84.26	99.76	85.36	72.79
Biorthogonal 1.5	84.47	99.76	85.93	73.12
Biorthogonal 2.4	**85.10**	**99.77**	**86.93**	**73.81**

The best experimental results are shown in bold.

#### 4.3.3 Ablation on wavelet fusion module

To investigate whether our designed wavelet fusion module facilitates the fusion of features from the dual-branch architecture, we conducted an ablation study. The results, as shown in Table 4, indicate that the model without the fusion block, where features from the two branches were simply element-wise added together in a “summation” operation, performed the worst with a Dice coefficient of only 79.58%. The performance of directly concatenating the feature maps from the two branches also lagged behind the model utilizing the wavelet fusion block. This suggests that our wavelet fusion module plays a significant role in enabling better information fusion and semantic alignment between the two feature maps, ultimately enhancing the model’s segmentation performance.

**TABLE 4:** Ablation study results on wavelet fusion module.

Methods	Dice (%)	Accuracy (%)	Sensitivity (%)	IoU (%)
Concatenation	81.23	99.72	80.56	70.21
Summation	79.58	99.71	79.64	70.07
Fusion Module	**85.10**	**99.77**	**86.93**	**73.81**

The best experimental results are shown in bold.

## 5 Discussion

Most existing networks for BVS segmentation in IVOCT images utilize the U-Net approach. This encoder-decoder structure effectively combines low-level detail information with high-level semantic information for segmentation tasks. However, current research has not adequately addressed issues such as detail loss in the downsampling process and artifacts in IVOCT images. A model that is robust to noise and anomalies is necessary to assist physicians in decision-making.

This study is inspired by the wavelet transform, a tool widely used in signal analysis known for its robustness to noise. After utilizing the 2D discrete wavelet transform, we can extract more detailed information from the original images. A feature fusion module is used to combine feature maps rich in semantic information with those rich in detail information for improved segmentation results.

Moreover, we addressed the issue of class imbalance due to the small area of stent struts relative to the entire image. We designed the Attention Gate (AG) and Atrous Multi-scale Field Module (AMFM) to enhance the model’s ability to capture foreground and background details while suppressing the response of irrelevant areas like the background. Overall, the dual-encoder structure proposed in this study leverages the advantages of wavelet transform and, through the AG and AMFM modules, enhances the model’s segmentation performance. However, our model has limitations. A dual-encoder increases the number of parameters, but this cost is justified as the credibility and robustness of an expert system are crucial for assisting physicians. Furthermore, if the detail information in the images is not significant, the enhancement from feature fusion may be minimal, thus not significantly improving segmentation results.

## 6 Conclusion

In this paper, we introduced a Wavelet-based U-shape Net for BVS segmentation in IVOCT images, effectively addressing the issue of detail loss during the downsampling process in traditional U-Net networks and enhancing the model’s segmentation performance. Firstly, by designing a wavelet-based dual-branch encoder, we enhanced the model’s capability to perceive details in feature extraction. Additionally, our designed feature fusion module supported the integration of feature maps from both branches. Secondly, through the Attention Gate (AG), we bolstered the model’s response to features in areas of interest, reducing false-positive segmentation. Finally, the atrous multiscale field module (AMFM) enables the model to learn more semantic contextual information, allowing it to capture information that is more useful for segmentation. Comparative experiments with other state-of-the-art models demonstrated that our model achieves the best performance. The results of ablation studies also validate the effectiveness of our designed modules. Through qualitative comparisons, our model better segments the detailed structures of stent struts in actual images. Considering the potential semantic disparity between feature maps from the two different branches, we plan to explore more diverse feature fusion methods for semantic alignment in the future, aiming for a more accurate integration of semantic and detail information. Additionally, given the time-consuming and labor-intensive nature of expert manual image annotation, we will also investigate training models using semi-supervised or unsupervised approaches. Finally, we will also explore the development of a BVSs segmentation model with increased robustness against noise, special stent conditions, and other scenarios in the future, to better assist clinical physicians in their treatment.

## Data Availability

The raw data supporting the conclusions of this article will be made available by the authors, without undue reservation.
