# Supplementary figures and images for: Wavelet-based U-shape network for bioabsorbable vascular stents segmentation in IVOCT images
Source: Front Physiol. 2024 Aug 15;15:1454835. doi: 10.3389/fphys.2024.1454835 (PMC11358552; doi:10.3389/fphys.2024.1454835)

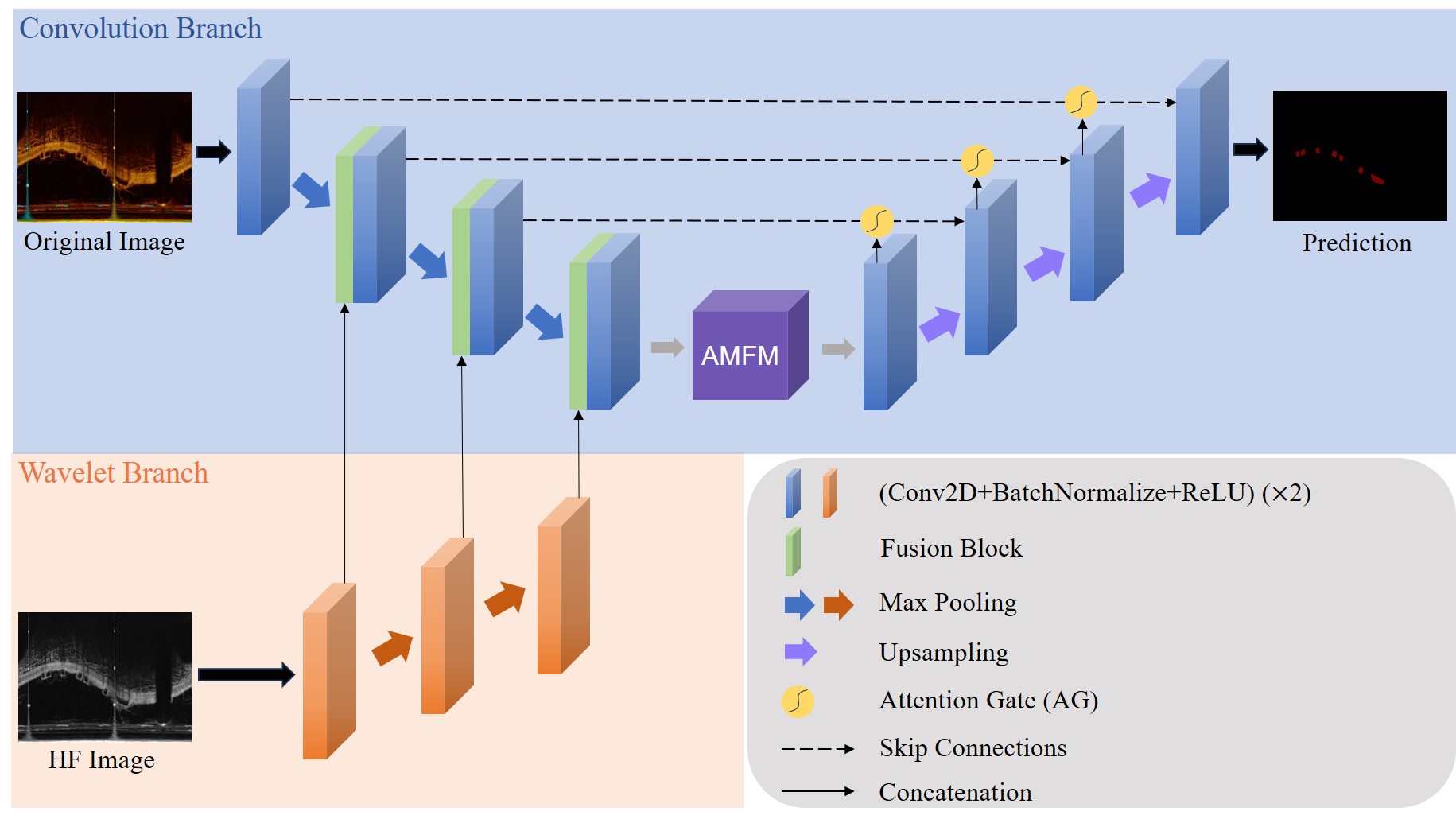

Supplement: Supplementary file 1 [file Image3.JPEG]

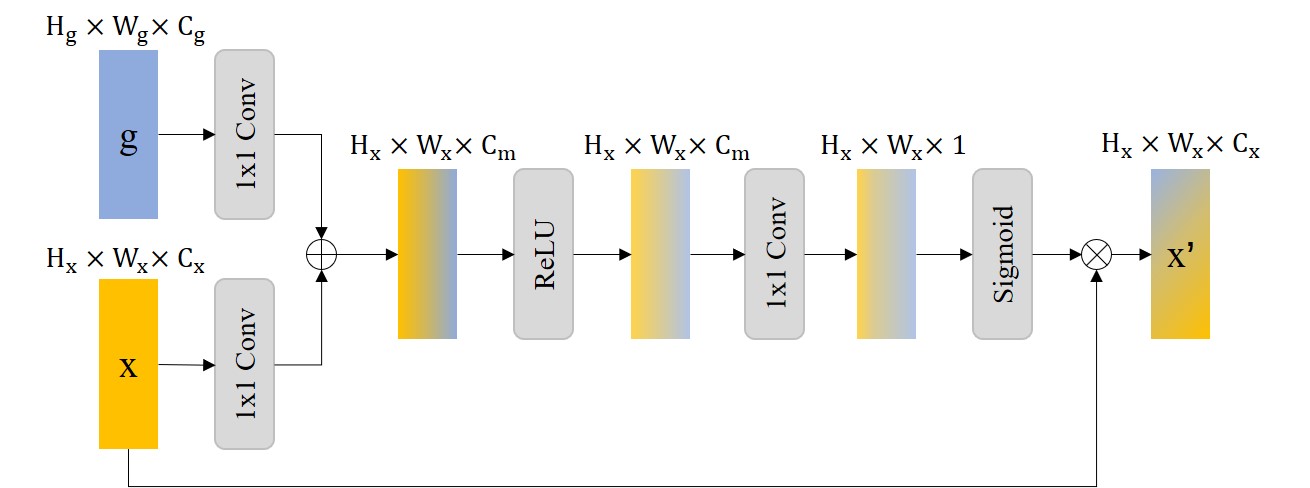

Supplement: Supplementary file 2 [file Image1.JPEG]

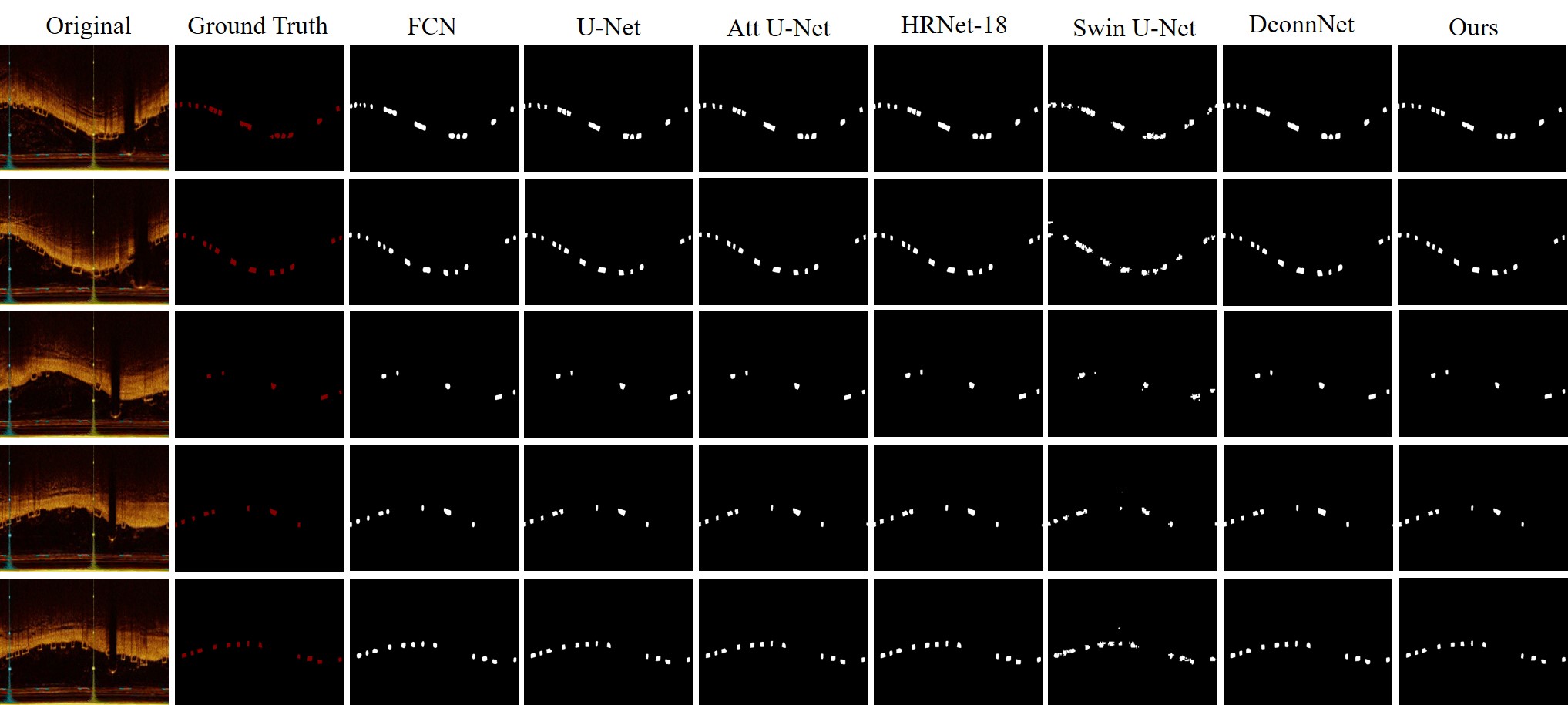

Supplement: Supplementary file 3 [file Image4.JPEG]

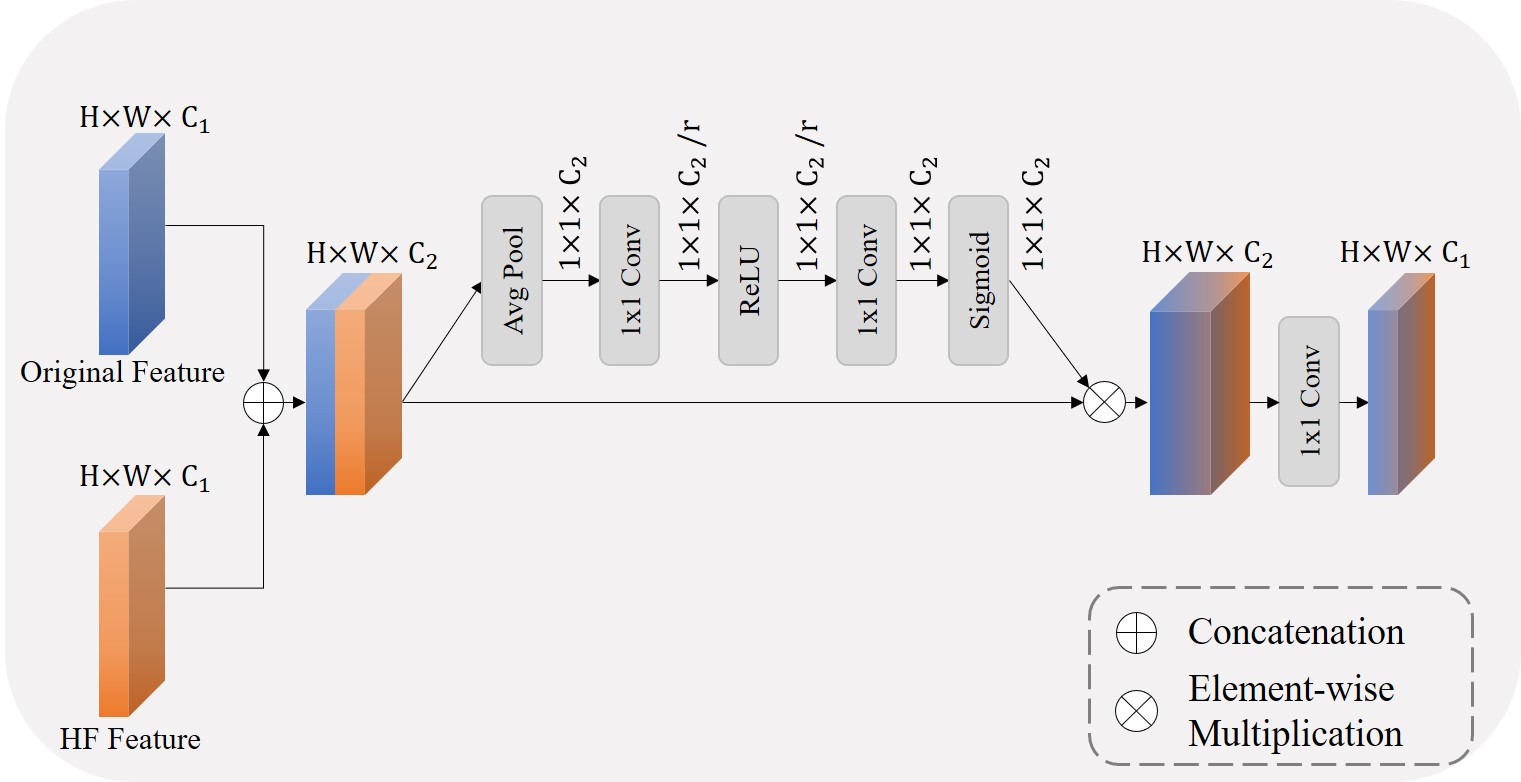

Supplement: Supplementary file 4 [file Image2.JPEG]

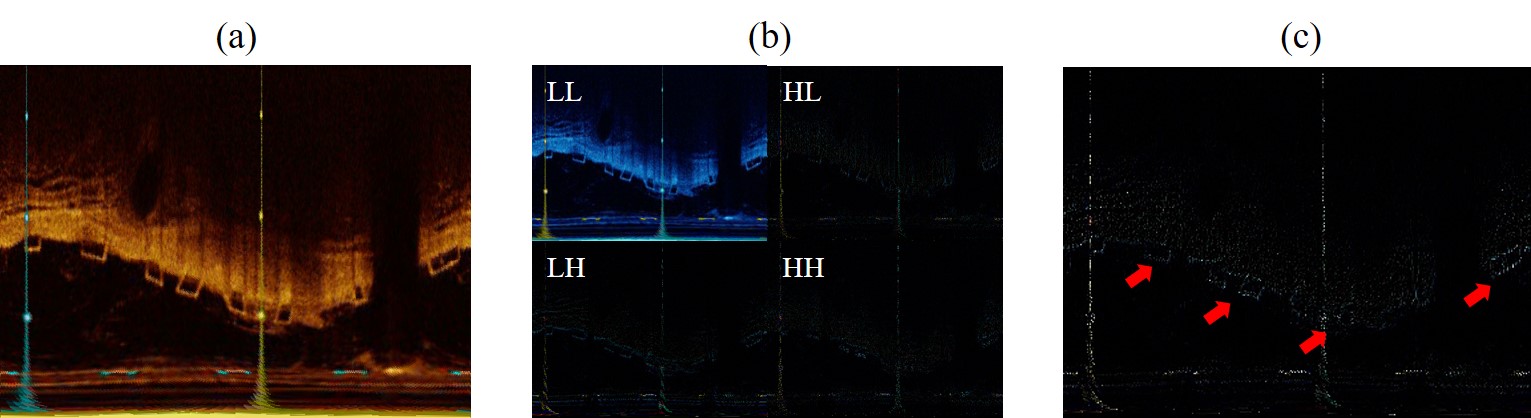

Supplement: Supplementary file 5 [file Image5.JPEG]

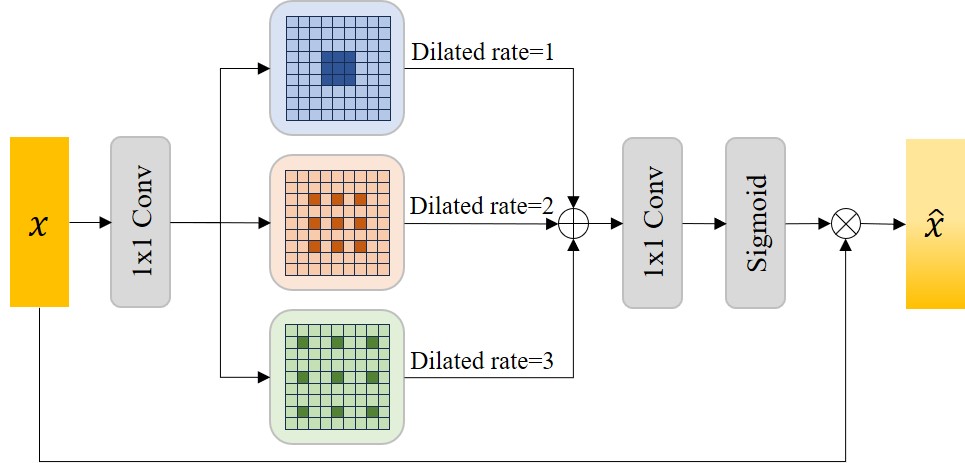

Supplement: Supplementary file 6 [file Image6.JPEG]
